# The Motivation of Medical Staff and the Work Interestedness in the Context of the COVID-19 Pandemic, in a Tertiary Hospital in Romania

**DOI:** 10.3390/healthcare11060813

**Published:** 2023-03-09

**Authors:** Codrin Dan Nicolae Ilea, Mădălina Diana Daina, Alina Cristiana Venter, Corina Lacramioara Șuteu, Monica Sabău, Dana Badau, Lucia Georgeta Daina

**Affiliations:** 1 Faculty of Medicine and Pharmacy, Doctoral School, University of Oradea, 410081 Oradea, Romania; 2 Faculty of Medicine and Pharmacy, University of Oradea, 410081 Oradea, Romania; 3 Department of Morphologycal Sciences, Faculty of Medicine and Pharmacy, University of Oradea, 410081 Oradea, Romania; 4 Department of Psycho-Neurosciences and Recovery, Faculty of Medicine and Pharmacy, University of Oradea, 410081 Oradea, Romania; 5 Petru Maior Faculty of Sciences and Letters, George Emil Palade University of Medicine, 540142 Targu Mures, Romania; 6 Interdisciplinary Doctoral School, Transilvania University, 500068 Brasov, Romania

**Keywords:** medical staff, motivation, work interestedness, tertiary hospital

## Abstract

The purpose of this study was to evaluate the impact of the COVID-19 pandemic on the motivation and work interestedness of employees in a tertiary hospital located in the northwest of Romania. In the study, 2230 employee satisfaction questionnaires distributed during 2019–2021 in the Oradea Emergency County Clinical Hospital (CCEHO) were analyzed. The percentage of those who declare themselves motivated at the hospital level remains relatively constant, at around 75%. There were differences between staff categories. The percentage of those who evaluate work interestedness with the qualifier “high” decreased from 45.45% for the year 2019 to 41.78% for the year 2021. The degree of motivation and work interestedness showed a significant increase for TESA staff during the pandemic period compared to the year 2019. A non-significant statistical increase in the percentage of motivated staff was observed among physicians, auxiliary staff and the radiology department staff. The motivation of nurses and laboratory staff decreased, but statistically insignificantly. The COVID-19 pandemic brought statistically significant changes at the level of motivation of the hospital staff only for the administrative staff, and the work interestedness for physicians and nurses decreased statistically significantly, especially in the second year of the pandemic. Older staff with an average level of education are more likely to be unmotivated, as shown by the regression model.

## 1. Introduction

In recent years, the health system in Romania has not benefited from the attention and funding necessary for a priority activity [1]. It faces frequent changes at a high level; in the period 2011–2021 there were 14 Ministers of Health and frequent changes in the management of the National Health Insurance House [2,3]. Even if, in 2018, there were significant salary increases for the staff in the state health system, these did not lead to an increase in the level of motivation for all categories of employees as expected [2,4]. At the end of 2019, the health system was not prepared to face the pandemic that would follow [1].

The first case of COVID-19 was registered in Romania on 26 February 2020 [5]. The state of emergency due to the COVID-19 pandemic was proclaimed on 16 March 2020 and maintained for 60 days [6]. This was followed by the alert status, which was maintained until 8 March 2022. Through ”Order no. 533/2020 on the approval of the Plan of measures for the preparation of hospitals in the context of the COVID-19 coronavirus epidemic and the List of support hospitals for patients tested positive for the SARS-CoV-2 virus”, published in the Official Gazette, Part I no. 263 of 31 March 2020, a list of measures was provided, among which we mention reducing the number of scheduled admissions by up to 80%, daily reporting of the number of occupied beds to the ministry, preparing hospitals for the admission of critically ill patients, increasing beds numbers in intensive care units, etc. [7]. Up to 23 December 2022, 3,305,048 people with COVID-19 were confirmed in Romania, of whom 67,341 died [8].

The health system and, implicitly, all medical and non-medical personnel were under enormous pressure. Limited knowledge and new information that was emerging every day led to a permanent need to adapt and find new solutions in the fight against the COVID-19 virus. As the number of cases of COVID-19 increased and the number of beds in hospitals, especially those in intensive care units, were insufficient, more and more shortcomings of the Romanian health system were revealed [9]. The first confirmed case of COVID-19 admitted to the Clinical County Emergency Hospital of Oradea (CCEHO) was on 17 May 2020. During 2020–2021, the hospital treated a total of 1614 confirmed patients with the SARS-CoV-2 virus. During the pandemic, CCEHO was classified as a second-line hospital.

During the analyzed period, the hospital had 861 beds in its structure, being a hospital of county interest that had addressability over a large area, providing emergency medical–surgical assistance in the territory for approximately 600,000 people. The hospital’s activity, in the period 2019 March 2020, involved the provision of diagnostic and therapeutic emergency medical services for an average of 350 patients per day, reaching an annual average of 130,000 patients, of which approximately 1/3 were hospitalized. After the declaration of the COVID-19 pandemic, organizational changes took place in the hospital to ensure emergency attention for ordinary cases but also for cases of SARS-CoV2 infection, with distinct functional circuits and rooms or wards dedicated to the treatment of patients infected with SARS-CoV2. This context also had implications for hospital employees, the first measures taken being the temporary suspension of paid leave and the designation of personnel directly involved in treating patients with COVID-19. By the end of 2021, approximately 80% of medical and auxiliary staff had cared for patients with COVID-19. Among the hospital employees, the technical-economic and administrative staff (TESA) were not involved in direct interaction with patients with COVID-19.

Previous studies have shown that infectious-contagious diseases such as COVID-19 or the Middle East respiratory syndrome coronavirus (MERS-CoV) are associated with a high degree of anxiety and stress among medical personnel, the main concern being the risk of disease transmission to the family and/or the risk of becoming sick themselves [10]. A meta-analysis published in October 2020 concluded that COVID-19 has a strong impact on the mental and physical health of healthcare workers [11]. A study carried out in Jordan has a similar conclusion [12]. The period of sanitary isolation led to the emergence of burnout syndrome and post-traumatic stress among health personnel. A study published in 2022 shows that 42% of family doctors in France suffered from psychological disorders [13]. A study conducted on medical and non-medical staff in a public hospital in Madrid shows a generalized and widespread psychological impact with no significant difference between medical and non-medical staff and no significant difference between front-line medical staff and the rest of the staff [14]. Another study conducted in Peru in 2022, among the staff of a district hospital, shows a strong correlation between moderate work motivation and a moderate form of depression [15]. A study conducted in two Romanian hospitals from March to June 2020 concluded that three job demands (work–family conflict, lack of preparedness/scope of practice, and emotional demands), three job resources (training, professional development, and continuing education; supervision, recognition, and feedback; autonomy and control), and one personal resource (self-efficacy) were significant predictors of burnout, explaining together 37% of the variance in healthcare workers’ burnout [16].

The analysis of employee motivation has played a central role in managerial theory and practice since the 20th century [17]. Theories and research on motivation in the work field proliferate significantly after 1965 [17,18]. In the early 1900s, it was believed that the main motivating factor for the employee was financial [19]. Of course, over time other aspects that influence motivation at work are discovered, so that at the beginning of the 21st century needs, personality, values, environment, behavior, affection and education are taken into account [17,20]. The healthcare system is a complex mechanism, where quality is conditioned by the individual performance of several actors working together, which means that individual performance does not necessarily correlate with quality care [21,22,23]. The motivation of healthcare personnel influences the quality of care and the behavior at work [21]. The interestedness in the work performed is related to the characteristics of the position, to the organizational attraction and to the conditions of the working environment [24,25,26]. In general, the work environment can be described as the place, conditions and surrounding factors in which the person carries out their activity [27].

We believe that it is important for any manager of a healthcare facility, public or private, to have information related to the degree of motivation and appreciation of the interestedness in the work performed by subordinate staff and, as much as possible, about the factors that influence them.

Assessing the impact of the COVID-19 pandemic on the employees of the Romanian healthcare system is imperative. The present study is such an attempt within the CCEHO by analyzing the data collected in the satisfaction questionnaire distributed to employees in the 2019-2021 period.

The purpose of this study is to analyze to what extent staff motivation and interestedness in the work performed has varied during the COVID-19 pandemic. Thus, we describe two hypotheses that we wish to dispute:

―– *H*_0_1: the degree of staff motivation is influenced by the COVID-19 pandemic;
―– *H*_a_1: the degree of staff motivation is not influenced by the COVID-19 pandemic;
―– *H*_0_2: the interestedness of work is influenced by the COVID-19 pandemic;
―– *H*_a_2: the interestedness of work is not influenced by the COVID-19 pandemic.

## 2. Materials and Methods

### 2.1. Study Design

An employee satisfaction questionnaire is distributed annually within the CCEHO. The CCEHO staff is composed of over 1500 employees, of which approximately 250 are doctors and approximately 800 are nurses. A number of approximately 1000 questionnaires are distributed annually. Each department and clinical section receives a number of questionnaires proportional to the existing staff. Of the questionnaires distributed during 2019–2021, 2386 were completed by employees. After eliminating the questionnaires with incomplete data, 2230 questionnaires remained in the analysis. The questionnaires were distributed in proportion to the number of hospital employees to the following staff categories: doctors, nurses, auxiliary staff, laboratory staff, staff from the radiology and imaging department and technical-economic and administrative staff (TESA).

The employee satisfaction questionnaire included a total of 23 questions, three of which were socio-economic (age, gender and level of education). In this article, the answers received to two questions were analyzed:

− are you motivated? (question 1);
− appreciate the interestedness of the work performed. (question 2).

The first question is a dichotomous one with two possible answers: YES and NO. The second one had three answer options: low, medium and high. For this question, the evolution of the “high” answers was compared to the rest of the answer options. 

Ethical approval was obtained from the Ethical Committee of County Clinical Emergency Hospital Oradea, Romania no. 25319/12.10.2018, and the present study was conducted in accordance with the Declaration of Helsinki.

### 2.2. Statistical Analysis

Statistical analysis was performed using the program R. Chi-square test (χ^2^) and Fisher’s test were used to determine statistical significance. The confidence interval was set at 95% and the statistical significance threshold was considered 0.05. Generalized nonlinear regression models were used to test the influence of socio-economic factors on the analyzed variables. The “backward selection” method was used to create the model.

### 2.3. Participants 

The distribution of the questionnaires analyzed by year is shown in Table 1. The annual distribution of the analyzed questionnaires for each staff group is presented in Table 2. Inclusion criteria: age 22–65 years, male and female gender, full completion of the questionnaire, employed by the CCEHO. 

## 3. Results

The distribution of the questionnaires analyzed by year was as follows:

The average age of the surveyed employees did not show statistically significant variation over time. Auxiliary staff, laboratory staff and TESA staff had a higher average age compared to doctors or nurses (Table 2).

**Figure 1:**
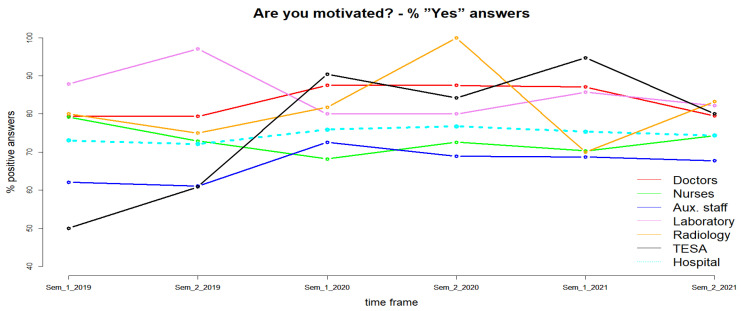
The evolution of the degree of motivation.

The percentage of those who declare themselves motivated at the hospital level remained relatively constant, at around 75% (Figure 1). An increase in the percentage of motivated people is observed from 72.60% for the year 2019 to 76.32% for the year 2020 and, respectively, to 74.89% for the year 2021. The observed difference is not statistically significant (χ^2^, *p* = 0.3). Doctors declare themselves motivated in a percentage of 79.37% for the year 2019, with a statistically insignificant increase (χ^2^, *p* = 0.3) to 87.5% during 2020 and, respectively, 83.43% for 2021 (Figure 1). For nurses, the pandemic period negatively influenced motivation, but the decrease observed from 76.06% (in 2019) to 70.30% (in 2020) and to 72.27% (in 2021) is not statistically significant (χ^2^, *p* = 0.3) (Figure 1). Similarly, for laboratory staff, the percentage of motivated people decreased from 92.54% for 2019 to 80% for 2020 and, respectively, to 83.93% for 2021 (χ^2^, *p* = 0.1).

For the auxiliary staff and those in the radiology department, the pandemic period did not bring statistically significant changes in terms of the degree of motivation. An increase in the percentage of motivated people was observed for the year 2020, followed by a decrease for the year 2021 but not below the percentage of the year 2019, a trend similar to that observed for physicians. Conversely, and surprisingly, TESA staff motivation increased from 55.56% in 2019 to 87.18% in 2021. The observed difference is statistically significant (χ^2^, *p* < 0.01). The motivation of CCEHO staff by professional category and gender of the respondent is presented in Table 3. At the hospital level it can be observed that the motivation of male staff increased more than that female staff in the 2020–2021 period. The difference in motivated people by gender for the year 2021 is statistically significant (χ^2^, *p* < 0.01). Female doctors had a higher degree of motivation than male doctors in 2020, but in 2021 the situation reversed (Table 3). Female nurses, representing the vast majority of the category, set the trend of decreasing motivation, as seen Figure 1.

**Figure 2:**
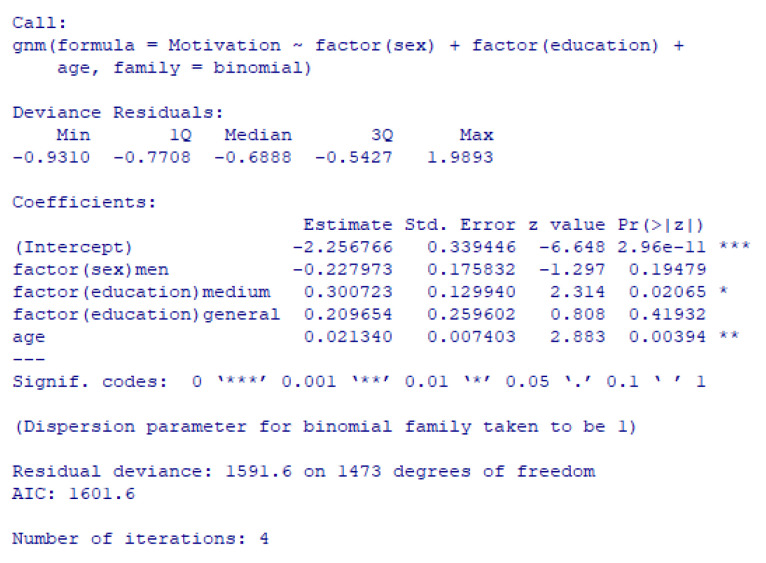
Generalized nonlinear regression model for motivation.

Similarly, the percentage of motivated women in the laboratory and in the radiology department was lower than that of men, but the differences were not statistically significant (Table 3). The motivation of auxiliary staff had a similar trend to that of physicians; thus, women were more motivated in 2020 and men were more motivated in 2021. Women in the TESA department were more motivated than men in 2019 and 2020, with a reverse situation for 2021, but the differences are not statistically significant (Table 3). Using a generalized non-linear regression model, we tested whether staff motivation was influenced by gender, age and level of education. The results show that motivation was statistically significantly influenced by age and education level, but not by gender (Figure 2). Older staff with an average level of education are more likely to be unmotivated (Figure 2). 

**Figure 3:**
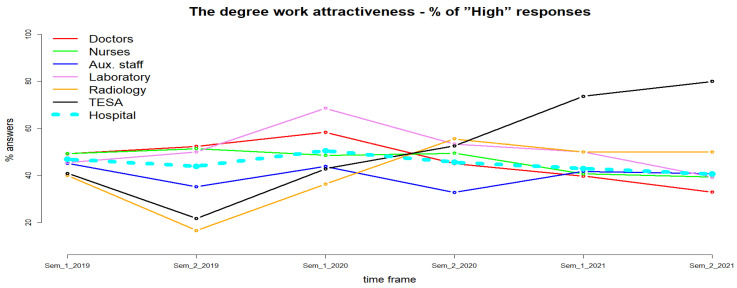
Work interestedness.

In the second analyzed question, employees were asked to rate the attractiveness of the work performed on a scale with three possible values: low, medium and high. Following the analysis of the questionnaires for the period 2019–2021, at the hospital level an increase was observed for the year 2020, from 45.45% (year 2019) to 48.27%, followed by a decrease in the year 2021 (41.78%) of those who appreciated the interestedness of the work performed as high (Figure 3). This annual fluctuation is statistically significant (χ^2^, *p* < 0.0001).

For doctors, the same trend is observed as for the entire hospital. An increase was observed for the year 2020 (52.27%), followed by a decrease for the year 2021 (36.46%) below the level of the year 2019 (50.79%). The observed difference is statistically significant (χ^2^, *p* < 0.0001). For nurses, the degree of work interestedness decreased slightly in 2020 (49.01%) compared to 2019 (50.35%) and by more than 10% in 2021 (40.06%) (χ^2^, *p* < 0.0001). The degree of job interestedness also decreased for auxiliary staff, from 40.52% in 2019 to 38.81% in 2020, but increased to 41.22% in 2021 (χ^2^, *p* = 0.9) (Figure 3). For laboratory staff, an increase in the degree of work interestedness was observed to 61.54% for the year 2020 compared to 47.76% for the year 2019, followed by a decrease to 44.64% for the year 2021, but without statistical significance (χ^2^, *p* = 0.1). For the staff in the radiology and imaging department there is an increasing trend, from 29.63% for the year 2019 to 45% for the year 2020 and 50% for the year 2021, in the degree of work interestedness, but still without statistical significance (χ^2^, *p* = 0.3). TESA staff rated work interestedness at a high level as follows: 31.11% in 2019, 47.50% in 2020 and 76.92% in 2021. The increase is statistically significant (χ^2^, *p* < 0.01) (Figure 3). Using a generalized nonlinear regression model, we tested whether the degree of work interestedness was influenced by gender, age and education level. The results show that they do not influence the monitored variable with any statistical significance.

## 4. Discussions

The degree of motivation of the CCEHO staff increased slightly in 2020 and 2021 compared to the pre-pandemic period, but without statistical significance. Thus, we can reject the null hypothesis 1 and implicitly accept the alternative hypothesis 1. The percentage of motivated staff among doctors, auxiliary staff and radiology department staff increased in 2020 above the value of 2019. This percentage decreased in 2021, but remained above the value of 2019. The variation is not statistically significant. The percentage of motivated doctors (83.43%) was higher than that of nurses (72.27%) in the second year of the pandemic. Similar results are presented by a study conducted in Pakistan from September 2020 to January 2021, showing that doctors and technicians have a higher motivation than nurses [28].

Among nurses, the percentage of those motivated decreased from 76.06% in the pre-pandemic period to 70.30% in the first year and, respectively, to 72.27% in the second year of the pandemic, but the variation is not statistically significant. The opposite results are described by various articles in the literature, showing an increase in nurse satisfaction during the COVID-19 pandemic. Thus, a study conducted at a clinical hospital in northern Portugal in 2022 concluded that the general satisfaction of nurses increased statistically significantly during the COVID-19 pandemic compared to the pre-pandemic period [29]. A high degree of job satisfaction is also present among nurses in Valencia (Spain), with values measured between 29 March and 8 April 2020 [30]. Nurses who cared for COVID-19 patients had lower job satisfaction compared to colleagues who did not care for confirmed patients [31,32]. This trend seemed to be maintained throughout the pandemic period. A study conducted at the beginning of the pandemic, 10–24 April 2020, in Zagazig (Egypt), and another conducted from 22 July to 16 November 2020 in Quebec (Canada), showed that nurses who cared for patients confirmed with COVID-19 had statistically significantly lower work satisfaction compared to colleagues who did not take care of such patients [31,32]. Unfortunately, our study did not have any clear information about which of the CCEHO medical and auxiliary staff surveyed were directly involved in treating patients with COVID-19 during the analyzed period and how long they cared for these patients.

Work satisfaction during the pandemic was lower among nurses in hospitals compared to colleagues in other healthcare units [33]. A greater workload among nurses due to staff shortages was not associated with decreased satisfaction [33]. A study conducted in Nepal showed that the job satisfaction of laboratory staff was negatively affected by the pandemic, decreasing from 67.4% before the pandemic to 43.19% during the pandemic [34]. Among the CCEHO laboratory staff, there was a decrease in the percentage of satisfied personal from 92.54% in the pre-pandemic period to 80% (year 2020) and 83.93% (year 2021); that is, however, not statistically significant (χ^2^, *p* = 0.1). This aspect can be attributed to the enormous workload, tests for detection of COVID-19 patients being analyzed practically non-stop in the first year of the pandemic. TESA staff were more motivated, statistically significantly, during the pandemic compared to 2019. The percentage of motivated TESA staff increased from 55.56% in 2019 to 87.18% in 2021.

Our study shows that the gender of the respondent influenced the degree of motivation, but it was statistically significant only for the hospital as a whole and only for the year 2021. Men were more motivated than women employed in the CCEHO. The generalized nonlinear regression model showed that motivation was negatively associated, statistically significantly, with older age and a medium level of education.

The work interestedness for CCEHO staff increased initially (year 2020) and then decreased (year 2021) below the value of 2019. This variation is statistically significant. Thus, we can accept null hypothesis 2, namely that work interestedness was affected by the COVID-19 pandemic. The work interestedness for doctors and nurses was influenced, statistically significant, in a negative way by the pandemic period. For the auxiliary staff, laboratory staff and radiology department staff, the variation in the percentage of people who rate the interestedness of the work as “high” did not change in the analyzed period in a statistically significant way. Instead, the percentage of TESA staff who rated the interestedness of the work as “high” showed a statistically significant increase for the pandemic period compared to the pre-pandemic period, from 31.11% in 2019 to 76.92% in 2021. Testing, with the help of the regression model, the influence of sex, age and level of education on the work interestedness variation shows us that there is no statistically significant association. A study conducted in private hospitals in northern India concluded that job satisfaction during the pandemic was not correlated with respondent’s gender, age, experience and marital status [35]. The study population included doctors, technical staff and auxiliary staff and was conducted between February and March 2021 [35]. Opposite results were presented by a study conducted in Jordan which concluded that job satisfaction was negatively associated with older age, stress/burnout, occupational category, low salary and a high activity workplace [12].

The form of the analyzed questionnaire, with yes and no questions, leads to some limitations in the interpretation of the results. Using a Likert scale questionnaire could lead to more nuanced results. The degree of staff motivation and work interestedness were analyzed using a single question. Furthermore, the results of the study are limited by the failure to include in the analysis some factors that could influence the degree of motivation and work interestedness, such as the treatment of confirmed cases, vaccination status, passing through illness, and family status.

The employee satisfaction questionnaire applied in the hospital, through the two analyzed items, allows the obtaining of an overview of the motivation and interestedness of work. To discover exactly what needs to be improved, it is necessary to design and apply specific questionnaires that correctly identify the factors involved in generating motivation and interestedness in the workplace. Additionally, taking into account the specifics of the activity of each workplace, department or clinic section, a comparative analysis by workplace is necessary.

## 5. Conclusions

The motivation of the CCEHO staff was not negatively influenced by the COVID-19 pandemic. On the contrary, there was a statistically insignificant increase in the percentage of motivated staff among doctors, auxiliary staff and those in the radiology department. The motivation of nurses and laboratory staff decreased, but statistically insignificantly. The work interestedness for doctors and nurses decreased statistically significantly, especially in the second year of the pandemic. TESA staff registered statistically significant increases both in the degree of motivation and the degree of work interestedness. The results of the study are limited by the failure to include in the analysis some factors that could influence the degree of motivation and work attractiveness, such as the treatment of confirmed cases, vaccination status, passing through illness, and family status. Additionally, using a Likert scale questionnaire could lead to more nuanced results.

## Figures and Tables

**Table 1:** Distribution by year, gender and education level of the analyzed questionnaires.

Year	2019	2020	2021	Total
No. of questionnaires	781	549	900	2230
Women %	82.84%	85.61%	80.56%	82.60%
Men %	17.16%	14.39%	19.44%	17.40%
Higher education %	47.98%	48.87%	51.41%	49.59%
Medium education %	44.24%	46.40%	42.95%	44.24%
General studies %	7.79%	4.73%	5.64%	6.17%

**Table 2:** Distribution by year and staff group of analyzed questionnaires and average age of staff.

Year	2019		2020		2021		Total	
Staff Employed	Nr. of Quest.	Average Age	Nr. of Quest.	Average Age	Nr. of Quest.	Average Age	Nr. of Quest.	AVERAGE Age
Physicians	126	43.48 ± 9.74	88	41.09 ± 11.79	181	42.91 ± 11.12	395	42.60 ± 10.89
Nurses	284	42.48 ± 7.64	202	44.46 ± 7.21	357	43.51 ± 8.34	843	43.38 ± 7.84
Aux. staff	232	46.22 ± 7.96	134	47.33 ± 7.58	245	45.04 ± 8.25	611	45.97 ± 8.04
Laboratory	67	46.27 ± 7.95	65	46.57 ± 8.13	56	46.51 ± 9.71	188	46.45 ± 8.54
Radiology	27	40.24 ± 10.20	20	46.61 ± 7.67	22	40.40 ± 11.32	69	42.11 ± 10.19
TESA	45	44.85 ± 6.89	40	48.48 ± 6.95	39	46.52 ± 7.55	124	46.45 ± 7.21
Hospital	781	44.07 ± 8.35	549	45.09 ± 8.62	900	44.10 ± 9.08	2230	44.34 ± 8.71

**Table 3:** The degree of staff motivation according to gender.

Professional Category	Sex	Year 2019			Year 2020			Year 2021		
		no.	% Yes	*p*	no.	% Yes	*p*	no.	% Yes	*p*
Physicians	women	70	77.14%	=0.6	47	89.36%	=0.8	110	79.09%	=0.08
	men	56	75.37%		41	85.37%		71	90.14%	
Nurses	women	272	75.37%	=0.3	195	69.23%	=0.2	329	72.34%	=1
	men	12	91.67%		7	100%		28	71.43%	
Aux. staff	women	192	60.94%	=0.8	118	72.03%	=0.6	195	65.64%	=0.1
	men	40	65%		16	62.50%		90	78%	
Laboratory	women	56	91.07%	=0.6	56	76.79%	=0.2	48	83.33%	=1
	men	11	100%		9	100%		8	87.50%	
Radiology	women	23	73.91%	=0.5	17	88.24%	=1	16	75%	=1
	men	4	100%		3	100%		6	83.33%	
TESA	women	34	58.82%	=0.7	37	89.19%	=0.8	27	81.48%	=0.3
	men	11	45.45%		3	66.67%		12	100%	
Hospital	women	647	71.72%	=0.3	470	75.11%	0.1	725	72.69%	<0.01
	men	134	76.87%		79	83.54%		175	84%	

*p*—level of probability, no.—number.

## References

[1] Constantin A., Mădălina-Gabriela A., Ștefan Virgil I. (2020). Study on the sanitary system in Romania. The stage and perspective of health evolution under the effect of the pandemic crisis (rou. Studiu privind sistemul sanitar din România. Stadiul şi perspectiva evoluţiei sănătăţii sub efectul crizei pandemice). Revista Română Statistica.

[2] (2021). OECD and European Observatory on Health Systems and Policies Romania: Country Health Profile. https://www.oecd-ilibrary.org/content/publication/74ad9999-en.

[3] (2021). Covid. Romania’s Health System Torn Apart by Pandemic—BBC News. https://www.bbc.co.uk/news/world-europe-58992090.

[4] Ilea C.D.N., Daina L.G., Bungau S., Tit D.M., Uivarosan D., Moleriu L., Petre I., Bungau C., Petre I. (2020). Sustainable Management, Instable Legislation Regarding Wages, and Employee Satisfaction/Motivation in Two Romanian Hospitals. Sustainability.

[5] Dascalu S. (2020). The Successes and Failures of the Initial COVID-19 Pandemic Response in Romania. Front. Public Health.

[6] Dascalu S., Geambasu O., Valentin Raiu C., Azoicai D., Damian Popovici E., Apetrei C. (2021). COVID-19 in Romania: What Went Wrong?. Front. Public Health.

[7] PLAN 29/03/2020—Legislative Portal. Just.ro. https://legislatie.just.ro/Public/DetaliiDocumentAfis/224.

[8] Mărcău F.C., Gheorghițoiu R., Bărbăcioru I.C. (2022). Survey upon the Reasons of COVID-19 Vaccination Acceptance in Romania. Vaccines.

[9] Lupu D., Tiganasu R. (2022). COVID-19 and the efficiency of health systems in Europe. Health Econ. Rev..

[10] Temsah M.-H., Al-Sohime F., Alamro N., Al-Eyadhy A., Al-Hasan K., Jamal A., Al-Maglouth I., Aljamaan F., Al Amri M., Barry M. (2020). The psychological impact of COVID-19 pandemic on health care workers in a MERS-CoV endemic country. J. Infect. Public Health.

[11] Salazar de Pablo G., Vaquerizo-Serrano J., Catalan A., Arango C., Moreno C., Ferre F., Shin J.I., Sullivan S., Brondino N., Solmi M. (2020). Impact of coronavirus syndromes on physical and mental health of health care workers: Systematic review and meta-analysis. J. Affect. Disord..

[12] Alrawashdeh H.M., Al-Tammemi A.B., Alzawahreh M.K., Al-Tamimi A., Elkholy M., Al Sarireh F., Abusamak M., Elehamer N.M.K., Malkawi A., Al-Dolat W. (2021). Occupational burnout and job satisfaction among physicians in times of COVID-19 crisis: A convergent parallel mixed-method study. BMC Public Health.

[13] Lange M., Joo S., Couette P.-A., Le Bas F., Humbert X. (2022). Impact on mental health of the COVID-19 outbreak among general practitioners during the sanitary lockdown period. Ir. J. Med. Sci..

[14] Leira-Sanmartin M., Madoz-Gurpide A., Ochoa-Mangado E., Ibáñez Á. (2021). Psychological impact of COVID-19 pandemic and related variables: A cross-sectional study in a sample of workers in a spanish tertiary hospital. Int. J. Environ. Res. Public Health.

[15] Xiao X., Zhu X., Fu S., Hu Y., Li X., Xiao J. (2020). Psychological impact of healthcare workers in China during COVID-19 pneumonia epidemic: A multi-center cross-sectional survey investigation. J. Affect. Disord..

[16] Cotel A., Golu F., Pantea Stoian A., Dimitriu M., Socea B., Cirstoveanu C., Davitoiu A.M., Jacota Alexe F., Oprea B. (2021). Predictors of Burnout in Healthcare Workers during the COVID-19 Pandemic. Healthcare.

[17] Toode K., Routasalo P., Suominen T. (2011). Work motivation of nurses: A literature review. Int. J. Nurs. Stud..

[18] Kanfer R., Frese M., Johnson R.E. (2017). Motivation related to work: A century of progress. J. Appl. Psychol..

[19] Ntoumanis N., Ng J.Y.Y., Prestwich A., Quested E., Hancox J.E., Thøgersen-Ntoumani C., Deci E.L., Ryan R.M., Lonsdale C., Williams G.C. (2021). A meta-analysis of self-determination theory-informed intervention studies in the health domain: Effects on motivation, health behavior, physical, and psychological health. Health Psychol. Rev..

[20] Latham G.P., Ernst C.T. (2006). Keys to motivating tomorrow’s workforce. Hum. Resour. Manag. Rev..

[21] Veenstra G.L., Dabekaussen K.F., Molleman E., Heineman E., Welker G.A. (2022). Health care professionals’ motivation, their behaviors, and the quality of hospital care: A mixed-methods systematic review. Health Care Manag. Rev..

[22] Gagn´e M. (2014). The Oxford Handbook of Work Engagement, Motivation, and Self-Determination Theory.

[23] Hollnagel E., Wears R.L., Braithwaite J. (2015). From Safety-I to Safety-II: A White Paper.

[24] Breaugh J.A. (2008). Employee recruitment: Current knowledge and important areas for future research. Hum. Resour. Manag. Rev..

[25] Burlea-Schiopoiu A., Baldo M.D., Idowu S.O. (2022). The Spirit of Adventure: A Driver of Interestedness of the Hospitality Industry for Young People during a Pandemic Crisis. Int. J. Environ. Res. Public Health.

[26] Åteg M., Hedlund A. (2011). Researching attractive work: Analyzing a model of attractive work using theories on applicant attraction, retention and commitment. Arbetsliv Omvandling.

[27] Wiskow C., Albreht T., De Pietro C. (2010). How to Create an Attractive and Supportive Working Environment for Health Professionals.

[28] Malik M., Rehan S.T., Malik F., Ahmed J., Fatir C.A., Ul Hussain H., Aman A., Tahir M.J. (2022). Factors associated with loss of motivation and hesitation to work amongst frontline health care providers during the COVID-19 pandemic: A cross-sectional survey from a developing country. Ann. Med. Surg..

[29] Ribeiro O.M.P.L., Coimbra V.M.O., Pereira S.C.d.A., Faria A.d.C.A., Teles P.J.F.C., Rocha C.G.d. (2022). Impact of COVID-19 on the Environments of Professional Nursing Practice and Nurses’ Job Satisfaction. Int. J. Environ. Res. Public Health.

[30] Gim´enez-Espert M.d.C., Prado-Gasco´ V., Soto-Rubio A. (2020). Psychosocial risks, work engagement, and job satisfaction of nurses during COVID-19 pandemic. Front. Public Health.

[31] Lavoie-Tremblay M., G´elinas C., Aub´e T., Tchouaket E., Tremblay D., Gagnon M.-P., Cˆot´e J. (2022). Influence of caring for COVID-19 patients on nurse’s turnover, work satisfaction and quality of care. J. Nurs. Manag..

[32] Said R.M., El-Shafei D.A. (2021). Occupational stress, job satisfaction, and intent to leave: Nurses working on front lines during COVID-19 pandemic in Zagazig City, Egypt. Environ. Sci. Pollut. Res. Int..

[33] Savitsky B., Radomislensky I., Hendel T. (2021). Nurses’ occupational satisfaction during Covid-19 pandemic. Appl. Nurs. Res..

[34] Basnet B.B., Satyal D., Pandit R., Maharjan A., Karki R., Mishra S.K., Gc S., Basnet T.B. (2022). Medical laboratory staff satisfaction and their perspective on the role of health institutions to combat COVID-19 pandemic. J. Int. Med. Res..

[35] Dixit V., Pant L.M., Chattopadhyay S. (2022). Perception of Work Satisfaction among Healthcare Workers during Covid-19 Pandemic at Private Hospitals of Bareilly District, UP. Specialusis Ugdymas.

